# Supplementary material for: (Sb0.5Li0.5)TiO3-Doping Effect and Sintering Condition Tailoring in BaTiO3-Based Ceramics
Source: Materials (Basel). 2024 Apr 29;17(9):2085. doi: 10.3390/ma17092085 (PMC11084442; doi:10.3390/ma17092085)
Supplement: Supplementary file 1 [file materials-17-02085-s001.zip › materials-2953128-supplementary.pdf]

Supporting information for

$(Sb_{0.5}Li_{0.5})TiO_3$ doping effect and sintering condition tailoring in $BaTiO_3$-based ceramics

Juanwen Yan[1], Bijun Fang[1,*], Shuai Zhang[1], Xiaolong Lu[1], Jianning Ding[1,2,*]

[1] School of Materials Science and Engineering, Jiangsu Collaborative Innovation Center of Photovoltaic Science and Engineering, Jiangsu Province Cultivation Base for State Key Laboratory of Photovoltaic Science and Technology, National Experimental Demonstration Center for Materials Science and Engineering, Changzhou University, Changzhou 213164, China

[2] School of Mechanical Engineering, Yangzhou University, Yangzhou 225127, China

* Corresponding authors.

E-mail addresses: fangbj@cczu.edu.cn (B. Fang), dingjn@cczu.edu.cn (J. Ding)

Tel.: +86 519 86330095; Fax: +86 519 86330095

Table S1. Energy storage performance of BSBiTZ-xSLT (x=0.025, 0.05, 0.075, 0.1) ceramics with the largest relative density at 50 kV/cm.

	P_{max} (μC/cm^2)	P_r (μC/cm^2)	E (kV/cm)	W_{rec} (mJ/cm^3)	W_t (mJ/cm^3)	η
x=0.025	2.7480	0.0383	50	65.59	67.61	97.02%
x=0.05	2.2171	0.0256	50	53.16	54.64	97.27%
x=0.075	1.9373	0.0601	50	44.94	48.37	92.90%
x=0.1	1.4218	0.0937	50	31.11	36.89	84.34%

Table S2. Charge/discharge performance of BSBiTZ-xSLT (x=0.025, 0.05, 0.075, 0.1) ceramics sintered at different temperatures at 30 kV/cm.

	Sintering temperature (°C)	E (kV/cm)	W_{dis} (mJ/cm^3)	I_{dis} (A)
	1125	30	33.43	9.25
	1140	30	32.78	9.40
x=0.025	1155	30	32.13	11.16
	1170	30	28.92	10.30
	1185	30	30.99	10.83
	1140	30	30.22	10.16
x=0.05	1155	30	24.00	10.01
	1170	30	26.27	10.67
	1140	30	19.06	8.79
x=0.075	1155	30	22.01	9.50
	1170	30	21.61	10.20
	1155	30	20.40	9.89
x=0.1	1170	30	16.46	9.40
	1185	30	17.83	9.69

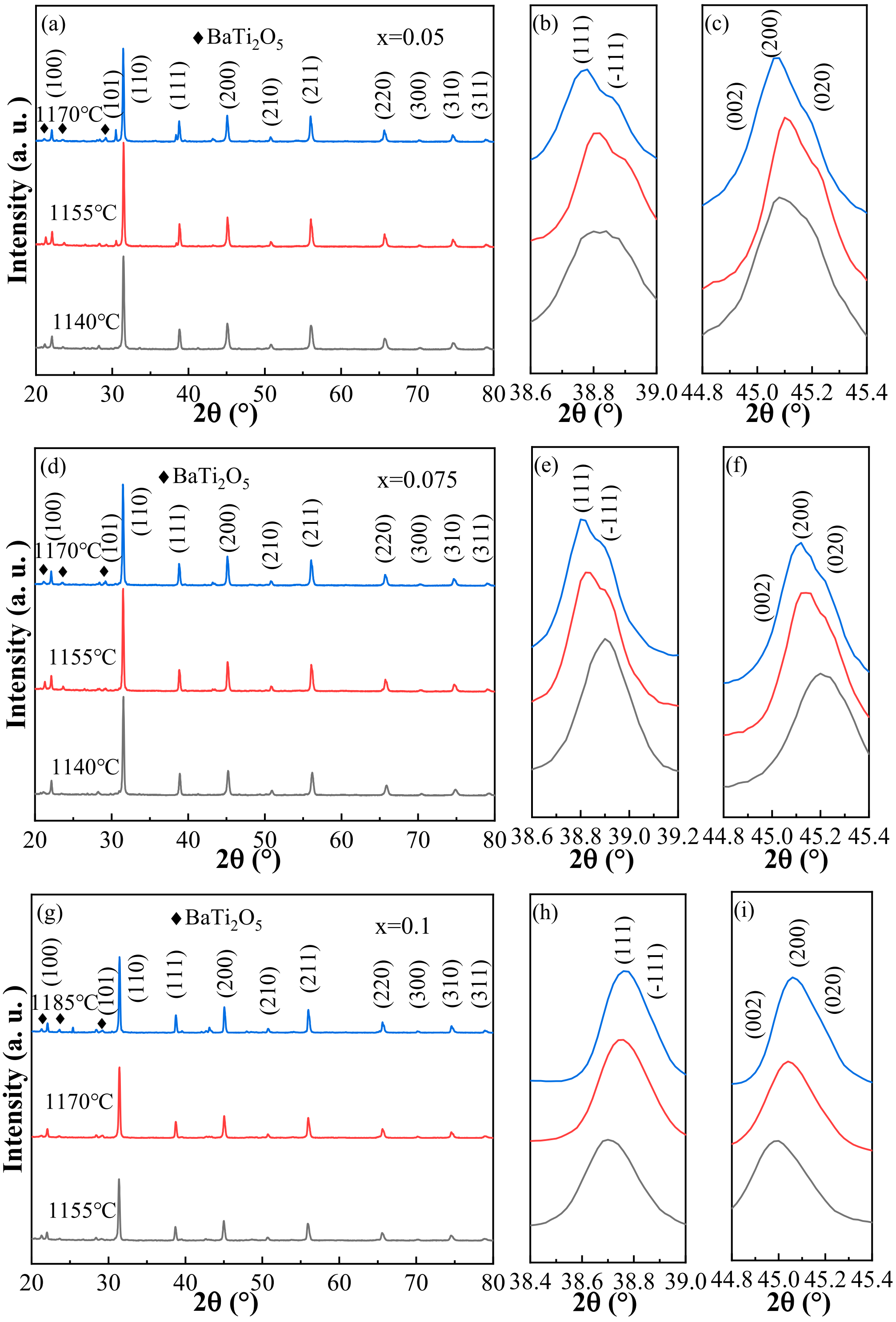

Figure S1. XRD patterns and enlarged XRD views of (111) and (200) diffraction peaks of BSBiTZ-xSLT (x=0.05, 0.075, 0.1) ceramics at different sintering temperatures. (a)-(c) BSBiTZ-0.05SLT; (d)-(f) SBiTZ-0.075SLT; (g)-(i) BSBiTZ-0.1SLT.

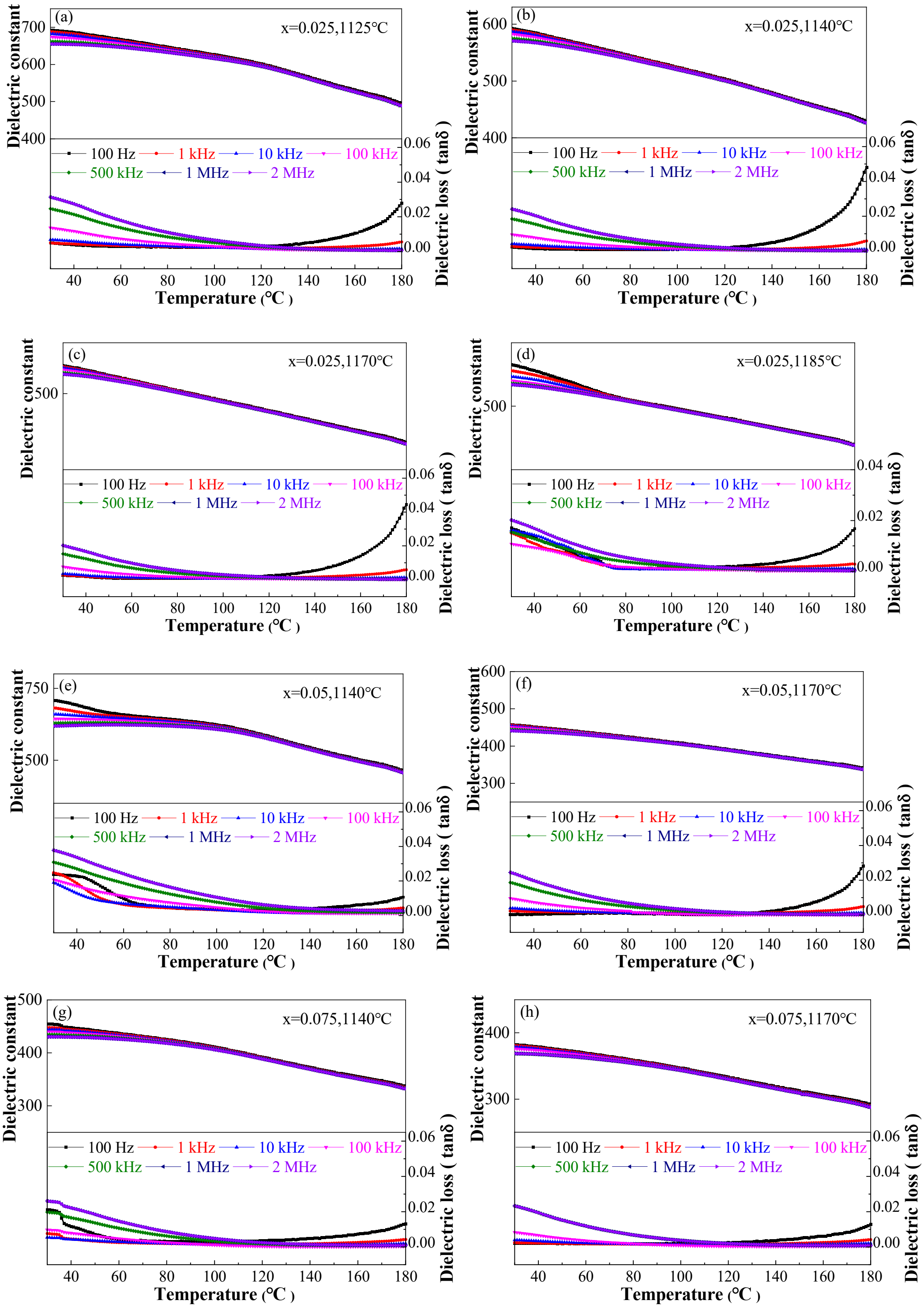

(a)
x=0.025,1125°C
(b)
x=0.025,1140°C
(c)
x=0.025,1170°C
(d)
x=0.025,1185°C
(e)
x=0.05,1140°C
(f)
x=0.05,1170°C
(g)
x=0.075,1140°C
(h)
x=0.075,1170°C
Dielectric constant
Dielectric loss (tanδ)
Temperature (°C)
100 Hz
1 kHz
10 kHz
100 kHz
500 kHz
1 MHz
2 MHz

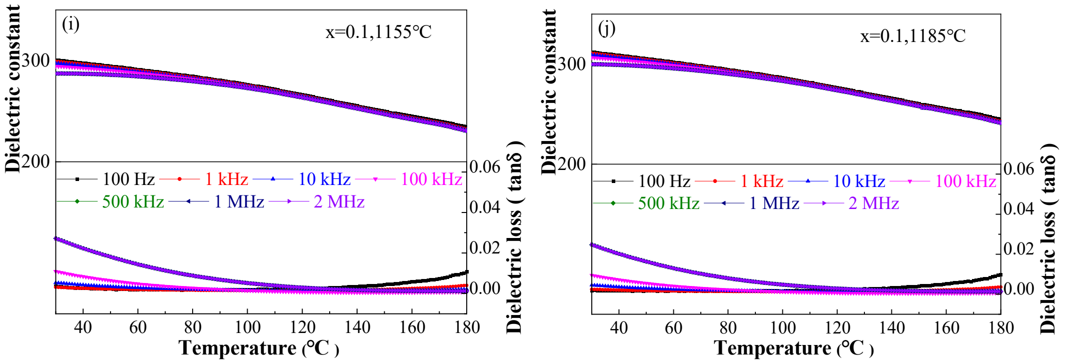

Figure S2. Dielectric-temperature curves of BSBiTZ-xSLT (x=0.025, 0.05, 0.075, 0.1) ceramics at different sintering temperatures. The composition of x and sintering temperature are shown in each figure.

(a)-(d) BSBiTZ-0.025SLT; (e)-(f) BSBiTZ-0.05SLT; (g) -(h) BSBiTZ-0.075SLT;

(i)-(j) BSBiTZ-0.1SLT.

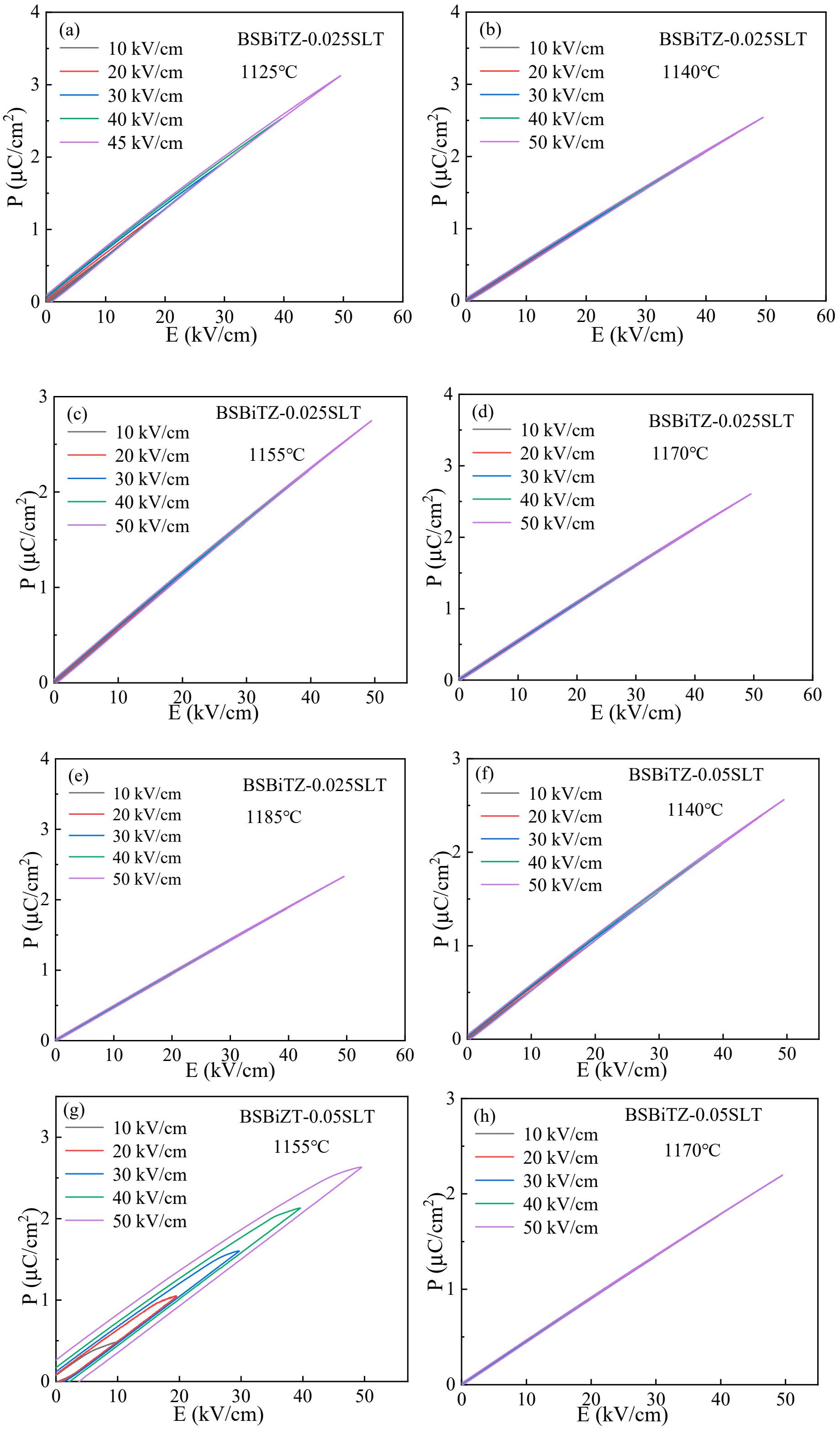

(a)
BSBiTZ-0.025SLT
1125℃
10 kV/cm
20 kV/cm
30 kV/cm
40 kV/cm
45 kV/cm
(b)
BSBiTZ-0.025SLT
1140℃
10 kV/cm
20 kV/cm
30 kV/cm
40 kV/cm
50 kV/cm
(c)
BSBiTZ-0.025SLT
1155℃
10 kV/cm
20 kV/cm
30 kV/cm
40 kV/cm
50 kV/cm
(d)
BSBiTZ-0.025SLT
1170℃
10 kV/cm
20 kV/cm
30 kV/cm
40 kV/cm
50 kV/cm
(e)
BSBiTZ-0.025SLT
1185℃
10 kV/cm
20 kV/cm
30 kV/cm
40 kV/cm
50 kV/cm
(f)
BSBiTZ-0.05SLT
1140℃
10 kV/cm
20 kV/cm
30 kV/cm
40 kV/cm
50 kV/cm
(g)
BSBiZT-0.05SLT
1155℃
10 kV/cm
20 kV/cm
30 kV/cm
40 kV/cm
50 kV/cm
(h)
BSBiTZ-0.05SLT
1170℃
10 kV/cm
20 kV/cm
30 kV/cm
40 kV/cm
50 kV/cm
P (μC/cm²)
E (kV/cm)

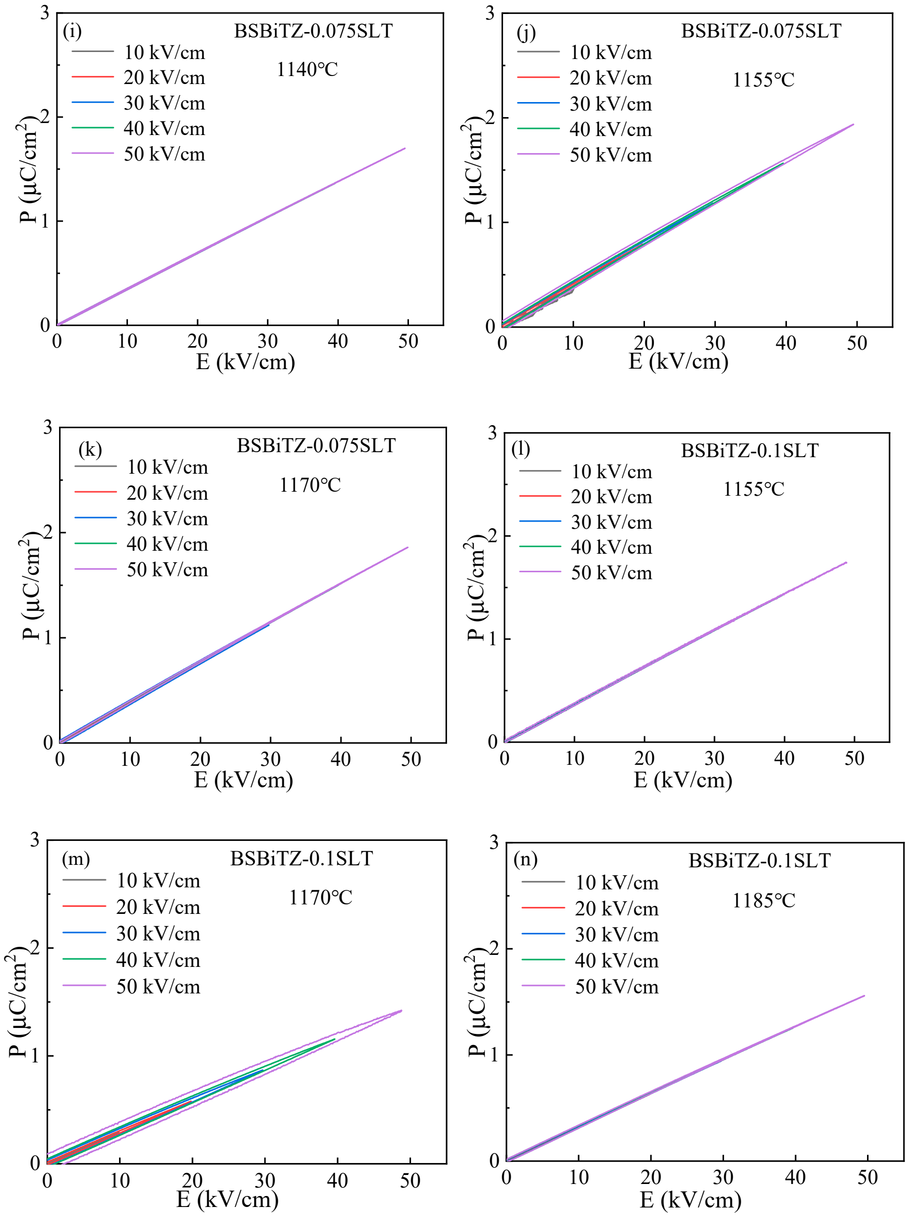

Figure S3. Unipolar hysteresis loops of BSBiTZ-xSLT (x=0.025, 0.05, 0.075, 0.1) ceramics at different sintering temperatures. The composition of x and sintering temperature are shown in each figure.

(a)-(e) BSBiTZ-0.025SLT; (f)-(h) BSBiTZ-0.05SLT; (i)-(k) BSBiTZ-0.075SLT;

(l)-(n) BSBiTZ-0.1SLT.

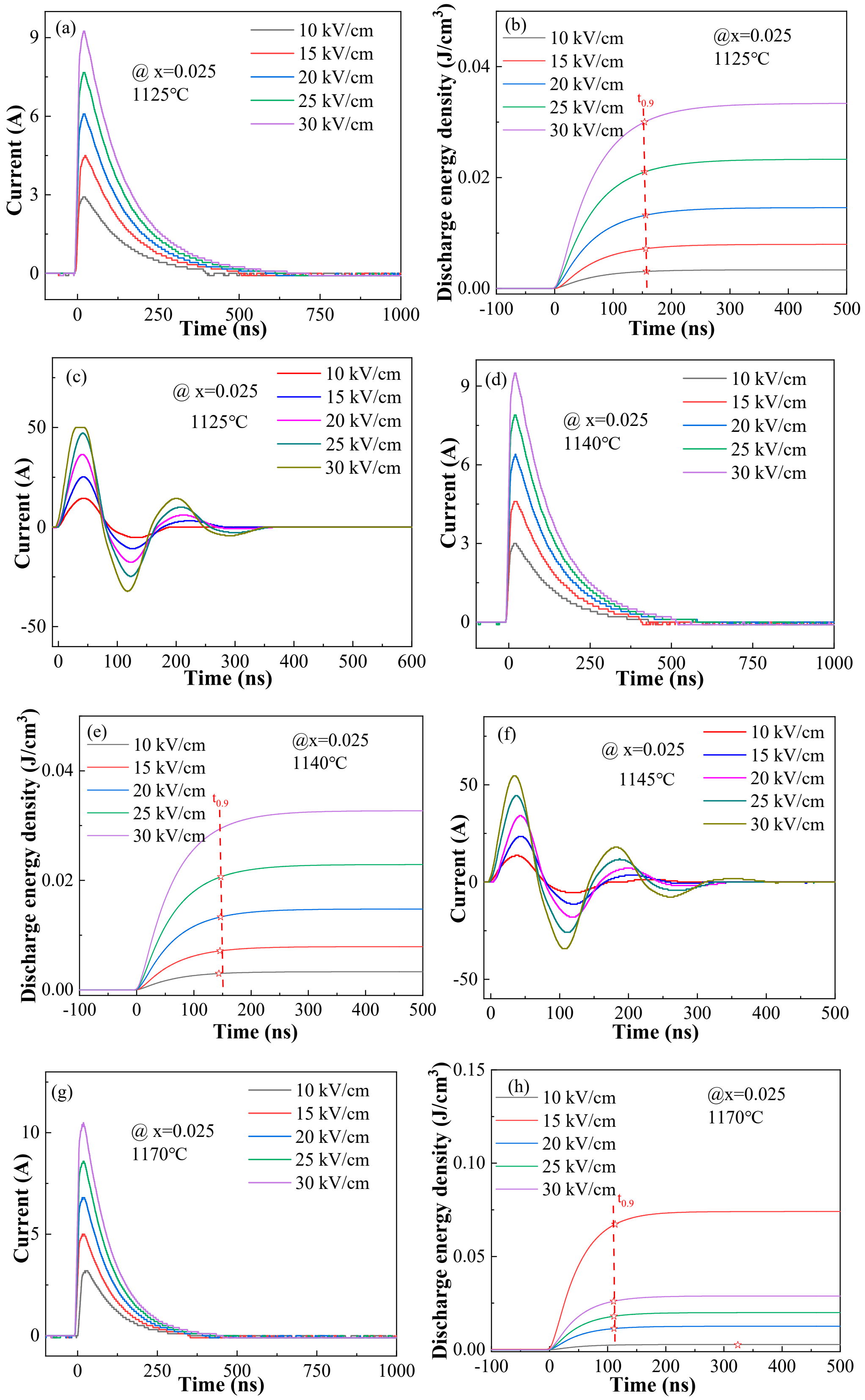

(a)
@ x=0.025
1125℃
10 kV/cm
15 kV/cm
20 kV/cm
25 kV/cm
30 kV/cm
Current (A)
Time (ns)
(b)
@x=0.025
1125℃
$t_{0.9}$
Discharge energy density (J/cm^3)
Time (ns)
(c)
@ x=0.025
1125℃
Current (A)
Time (ns)
(d)
@ x=0.025
1140℃
Current (A)
Time (ns)
(e)
@x=0.025
1140℃
$t_{0.9}$
Discharge energy density (J/cm^3)
Time (ns)
(f)
@ x=0.025
1145℃
Current (A)
Time (ns)
(g)
@ x=0.025
1170℃
Current (A)
Time (ns)
(h)
@x=0.025
1170℃
$t_{0.9}$
Discharge energy density (J/cm^3)
Time (ns)

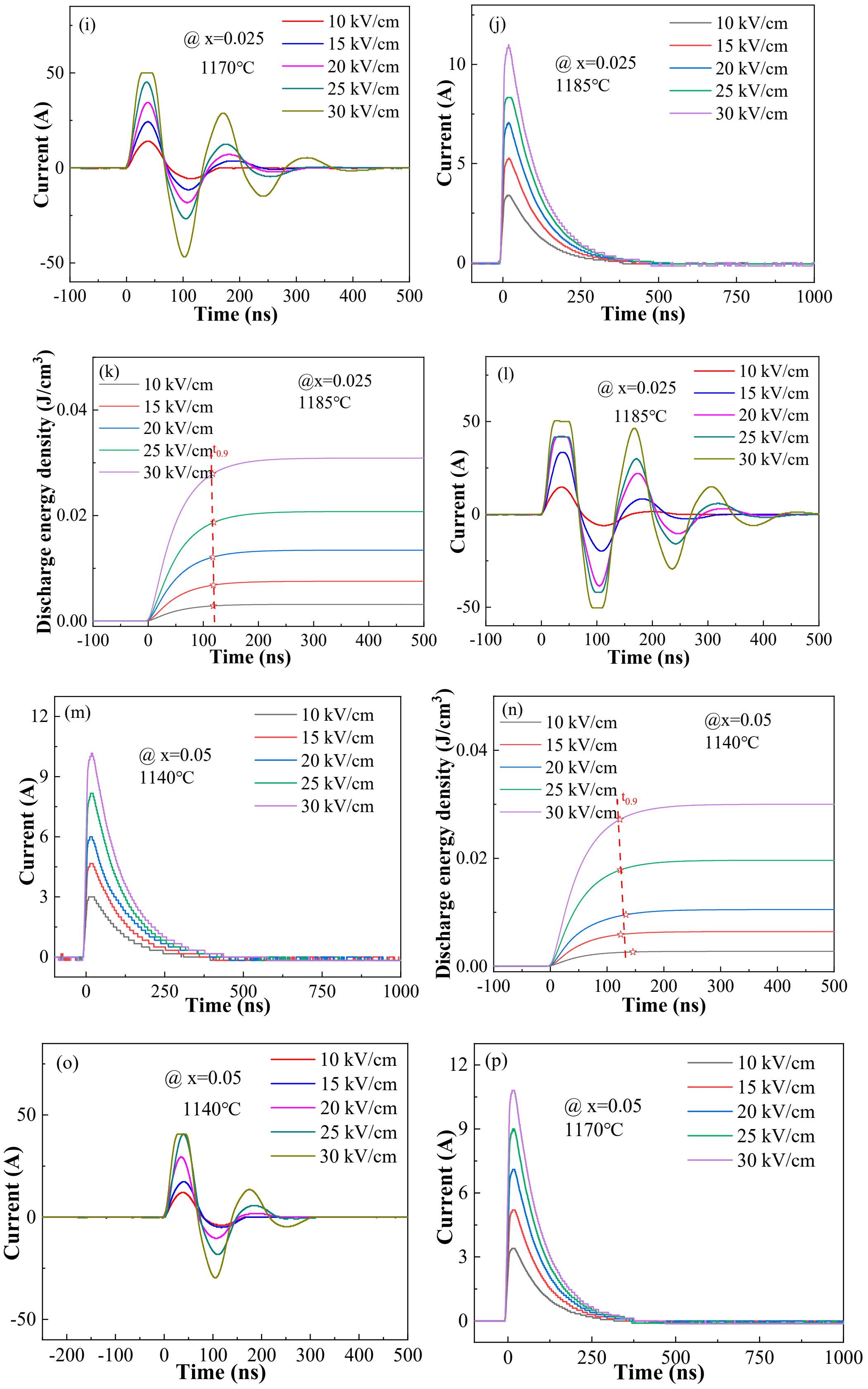

(i)
@ x=0.025
1170℃
10 kV/cm
15 kV/cm
20 kV/cm
25 kV/cm
30 kV/cm
Current (A)
Time (ns)
(j)
@ x=0.025
1185℃
10 kV/cm
15 kV/cm
20 kV/cm
25 kV/cm
30 kV/cm
Current (A)
Time (ns)
(k)
@x=0.025
1185℃
10 kV/cm
15 kV/cm
20 kV/cm
25 kV/cm
30 kV/cm
$t_{0.9}$
Discharge energy density (J/cm^3)
Time (ns)
(l)
@ x=0.025
1185℃
10 kV/cm
15 kV/cm
20 kV/cm
25 kV/cm
30 kV/cm
Current (A)
Time (ns)
(m)
@ x=0.05
1140℃
10 kV/cm
15 kV/cm
20 kV/cm
25 kV/cm
30 kV/cm
Current (A)
Time (ns)
(n)
@x=0.05
1140℃
10 kV/cm
15 kV/cm
20 kV/cm
25 kV/cm
30 kV/cm
$t_{0.9}$
Discharge energy density (J/cm^3)
Time (ns)
(o)
@ x=0.05
1140℃
10 kV/cm
15 kV/cm
20 kV/cm
25 kV/cm
30 kV/cm
Current (A)
Time (ns)
(p)
@ x=0.05
1170℃
10 kV/cm
15 kV/cm
20 kV/cm
25 kV/cm
30 kV/cm
Current (A)
Time (ns)

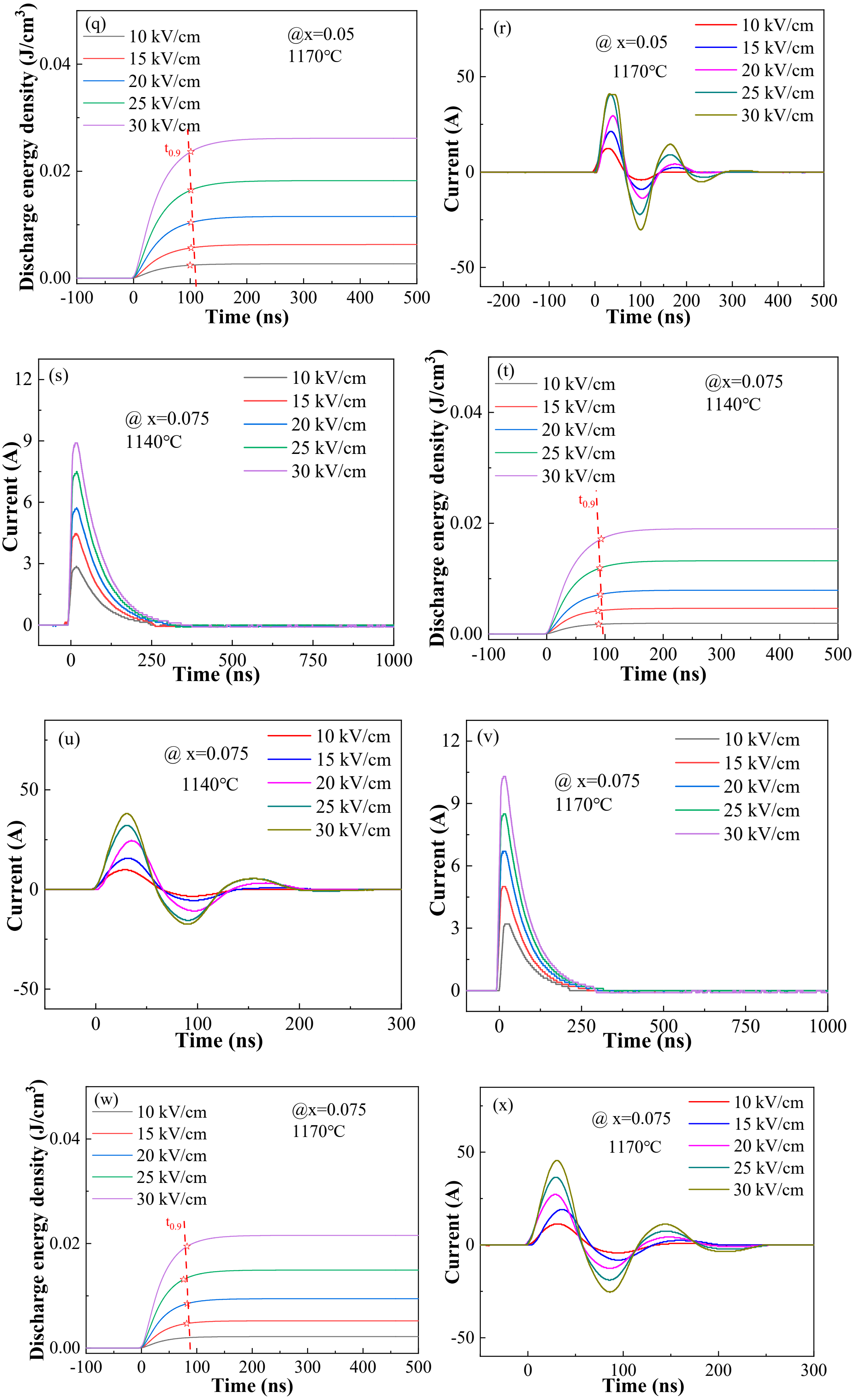
(q)
10 kV/cm
15 kV/cm
20 kV/cm
25 kV/cm
30 kV/cm
@x=0.05
1170℃
t0.9
Discharge energy density (J/cm3)
0.00
0.02
0.04
-100
0
100
200
300
400
500
Time (ns)
(r)
@ x=0.05
1170℃
10 kV/cm
15 kV/cm
20 kV/cm
25 kV/cm
30 kV/cm
Current (A)
50
0
-50
-200
-100
0
100
200
300
400
500
Time (ns)
(s)
@ x=0.075
1140℃
10 kV/cm
15 kV/cm
20 kV/cm
25 kV/cm
30 kV/cm
Current (A)
12
9
6
3
0
0
250
500
750
1000
Time (ns)
(t)
10 kV/cm
15 kV/cm
20 kV/cm
25 kV/cm
30 kV/cm
@x=0.075
1140℃
t0.9
Discharge energy density (J/cm3)
0.04
0.02
0.00
-100
0
100
200
300
400
500
Time (ns)
(u)
@ x=0.075
1140℃
10 kV/cm
15 kV/cm
20 kV/cm
25 kV/cm
30 kV/cm
Current (A)
50
0
-50
0
100
200
300
Time (ns)
(v)
@ x=0.075
1170℃
10 kV/cm
15 kV/cm
20 kV/cm
25 kV/cm
30 kV/cm
Current (A)
12
9
6
3
0
0
250
500
750
1000
Time (ns)
(w)
10 kV/cm
15 kV/cm
20 kV/cm
25 kV/cm
30 kV/cm
@x=0.075
1170℃
t0.9
Discharge energy density (J/cm3)
0.04
0.02
0.00
-100
0
100
200
300
400
500
Time (ns)
(x)
@ x=0.075
1170℃
10 kV/cm
15 kV/cm
20 kV/cm
25 kV/cm
30 kV/cm
Current (A)
50
0
-50
0
100
200
300
Time (ns)

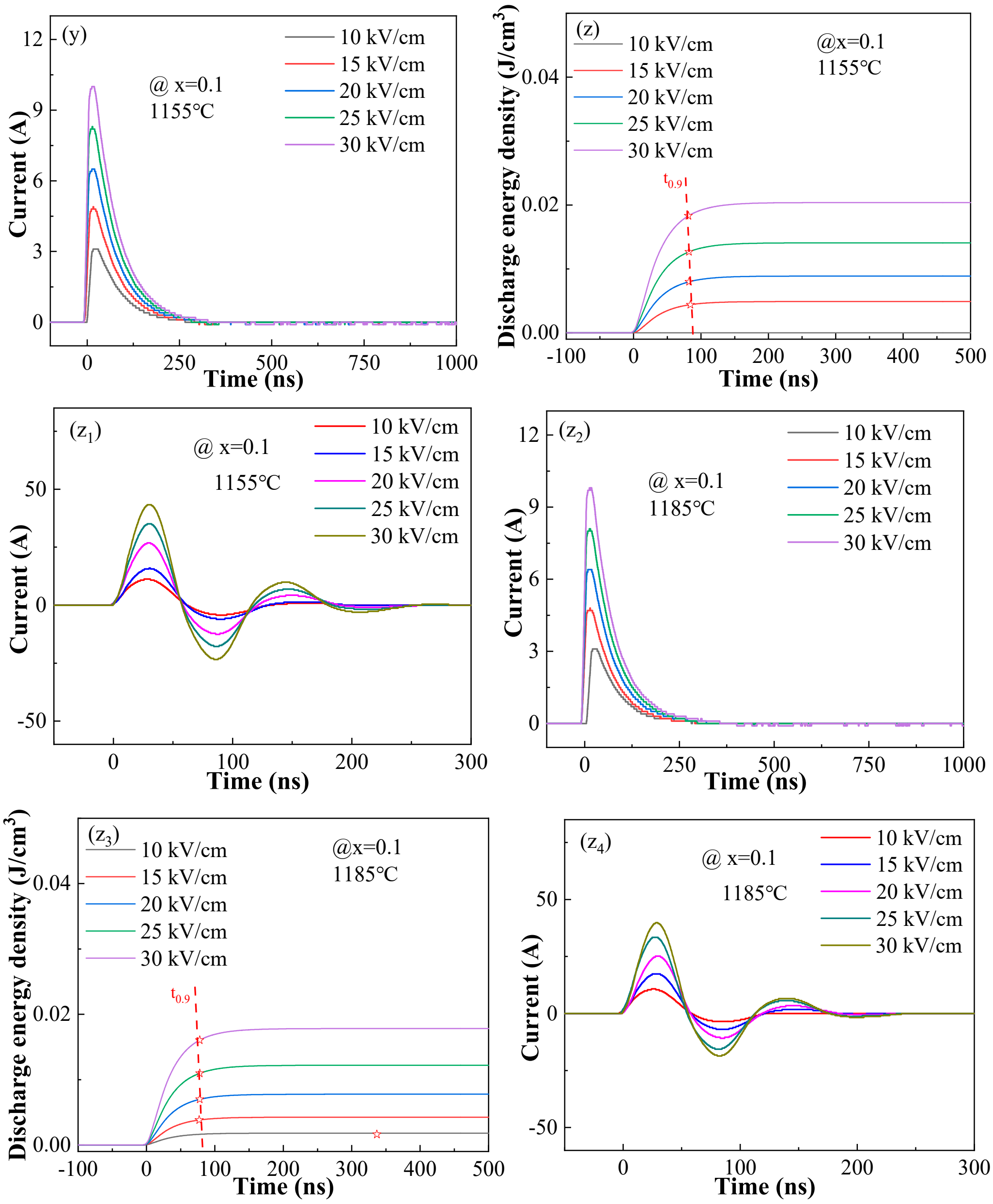

Figure S4. The pulse charge-discharge energy storage properties of BSBiTZ-xSLT (x=0.025, 0.05, 0.075, 0.1) ceramics at different sintering temperatures. The composition of x and sintering temperature are shown in each figure.

(a) Overdamped discharged voltage curve of BSBiTZ-0.025SLT at 1125 °C; (b) The variation of discharge energy density and $t_{0.9}$ with time of BSBiTZ-0.025SLT at 1125 °C; (c) Underdamped discharged voltage curve of BSBiTZ-0.025SLT at 1125 °C; (d)-(f) BSBiTZ-

0.025SLT at 1140 °C; (g)-(i) BSBiTZ-0.025SLT at 1170 °C; (j)-(l) BSBiTZ-0.025SLT at 1185 °C; (m)-(o) BSBiTZ-0.05SLT at 1140 °C; (p)-(r) BSBiTZ-0.05SLT at 1170 °C; (s)-(u) BSBiTZ-0.075SLT at 1140 °C; (v)-(x) BSBiTZ-0.075SLT at 1170 °C; (y)-(z_1) BSBiTZ-0.1SLT at 1155 °C; (z_2)-(z_4) BSBiTZ-0.1SLT at 1185 °C.
